# Performance of an Autonomous Sanitary Sterilisation Ultraviolet Machine (ASSUM) on terminal disinfection of surgical theaters and rooms of an intensive-intermediate care unit

**DOI:** 10.1016/j.infpip.2024.100396

**Published:** 2024-08-29

**Authors:** Sabina Herrera, Ignasi Roca, Ana Del Río, Javier Fernández, Cristina Pitart, Isabel Fortes, Blanca Torralbo, Gemina Santana, Romina Parejo-González, Andreu Veà-Baró, Josep Maria Campistol, Mireia Aguilar, Sergi Degea, Climent Casals-Pascual, Alex Soriano, José A. Martínez

**Affiliations:** a Infectious Disease Service, Hospital Clínic, University of Barcelona, IDIBAPS, Barcelona, Spain; b CIBER de Enfermedades Infecciosas (CIBERINFEC), Instituto de Salud Carlos III, Madrid, Spain; c Department of Microbiology, Biomedical Diagnostic Center (CDB) and ISGlobal, Hospital Clínic, University of Barcelona, Barcelona, Spain; d Liver ICU, Liver Unit, Hospital Clinic, University of Barcelona, IDIBAPS and CIBERehd, Spain; e EF Clif, EASL-CLIF Consortium, Barcelona, Spain; f CovidWarriors, Barcelona, Spain; g Preventive Medicine Service, Hospital Clinic, University of Barcelona, IDIBAPS, Barcelona, Spain; h Andreu Veà, Ph.D. advisor to the CEO (on Digital-Transformation & Optimization) Hospital Clinic Barcelona CovidWarrior, Barcelona, Spain; i Hospital Clínic, University of Barcelona, August Pi i Sunyer Biomedical Research Institute Barcelona, Spain

**Keywords:** Ultraviolet- C (UV–C), UVC robot, (MDR) microorganisms

## Abstract

**Background:**

Ultraviolet- C (UV–C) light is effective for reducing environmental bioburden in hospitals, and the use of robots to deliver it may be advantageous.

**Aim:**

To evaluate the feasibility and clinical efficacy of an autonomous programmable UV-C robot in surgical and intensive care unit (ICU) rooms of a tertiary hospital.

**Method:**

During ten consecutive months, the device was used in six theatres where cardiac, colorectal and orthopaedic surgeries were performed, and in the rooms previously occupied by patients subjected to contact precautions of a 14-bed ICU. Surgical site infection (SSI) rates of procedures performed in the UV-cleaned theatres were compared with those of the previous year. Incidence in clinical samples of ICU-acquired multiple-drug resistant (MDR) microorganisms was compared with that of the same period of the previous year. An UV-C exposure study done by semi-quantitative dosimeters and a survey of the bioburden on surfaces were carried out.

**Findings:**

SSI rates in the pre- and post-intervention periods were 8.67% (80/922) and 7.5% (61/813), respectively (p=0.37). Incidence of target microorganisms in clinical samples remained unchanged (38.4 vs. 39.4 per 10,000 patient-days, p=0.94). All the dosimeters exposed to ≤1 meter received ≥500 mJ/cm^2^. The bacterial load on surfaces decreased after the intervention, particularly in ICU rooms (from 4.57±7.4 CFU to 0.27±0.8 CFU, p<0.0001).

**Conclusion:**

Deployment of an UV-C robot in surgical and ICU rooms is feasible, ensures adequate delivery of germicidal UV-C light and reduces the environmental bacterial burden. Rates of surgical site infections or acquisition of MDR in clinical samples of critically-ill patients remained unchanged.

## Introduction

In order to prevent nosocomial infections, the promotion of environmental hygiene has a prominent role [1]. Among the procedures aimed at reducing the microbial load on surfaces, objects and air in the hospital, the application of germicidal ultraviolet light (UVL), usually as a complement to ordinary manual cleaning and disinfection, has raised a considerable interest in the last fifteen years [2,3]. UVL has been used for the terminal disinfection of operating theatres [[4], [5], [6], [7], [8], [9], [10]] and other hospital rooms, particularly those in high-risk areas or previously occupied by patients carrying multiple-drug resistant (MDR) microorganisms or *Clostridioides difficile* [[11], [12], [13], [14], [15], [16], [17]].

In terms of clinical efficacy, germicidal UVL has significantly reduced the overall rates of healthcare-related infections, as well as specifically those caused by *Clostridioides difficile*, vancomycin-resistant enterococci (VRE), methicillin-resistant *Staphylococcus aureus*, multiple-drug resistant Gram-negative bacilli [1,[11], [12], [13], [14], [15], [16], [17]] or Gram-negative bacilli bacteraemia [18]. Despite the overall favourable effects of germicidal UVL, there are still doubts about its efficacy due to the relatively low number of controlled studies. In one of the two randomized trials already published, efficacy was limited to *C. difficile* and VRE [19], while in the other no effect was observed on the incidence of these microorganisms in four oncology and one solid organ transplant wards [20].

The germicidal action of UVL depends on the dose delivered to the surface that, in turn, is related to the irradiance produced by the device, to the angle of incidence and to the surface being in the direct line of sight or in areas of shadow in relation to the emitting source [2,3,11,17]. Different microorganisms may show different susceptibilities; *C. difficile* spores, for example, are among the most resistant bacteria [2,11,17]. In order to ensure the correct exposure of surfaces, it is common to have to move the emitting source or deploy more than one device in the room. An innovative strategy to optimize the exposition has been the installation of the UVL emitter on automotive robots. These instruments allow the programming of the most appropriate routes in terms of distance and irradiation times for optimising the exposure of any surface within the space to be disinfected [21,22]. Although the germicidal efficacy of autonomous UV robots has been proven against bacteria, fungi and viruses [[21], [22], [23]], their feasibility of operation and clinical effectiveness in terms of reducing healthcare-related infections has not yet been widely demonstrated in complex health institutions such as tertiary acute hospitals [24,25].

Therefore, there is a need for more controlled studies to conclusively determine the clinical efficacy of germicidal UVL, particularly in complex healthcare environments. So far, mixed results from existing RCTs indicate the necessity to explore the efficacy of UVL in diverse clinical settings and for different pathogens. Although strategies like using UVL-emitting robots have shown promise, there is a lack of comprehensive evaluation of their operational feasibility and clinical effectiveness in tertiary acute hospitals.

The present research aims to evaluate the on-the-field feasibility and clinical efficacy of an autonomous programmable ultraviolet light- C (UV–C) light emitting robot in a tertiary academic hospital.

## Methods

### Technical description of the device and place of study

**Figure 1:**
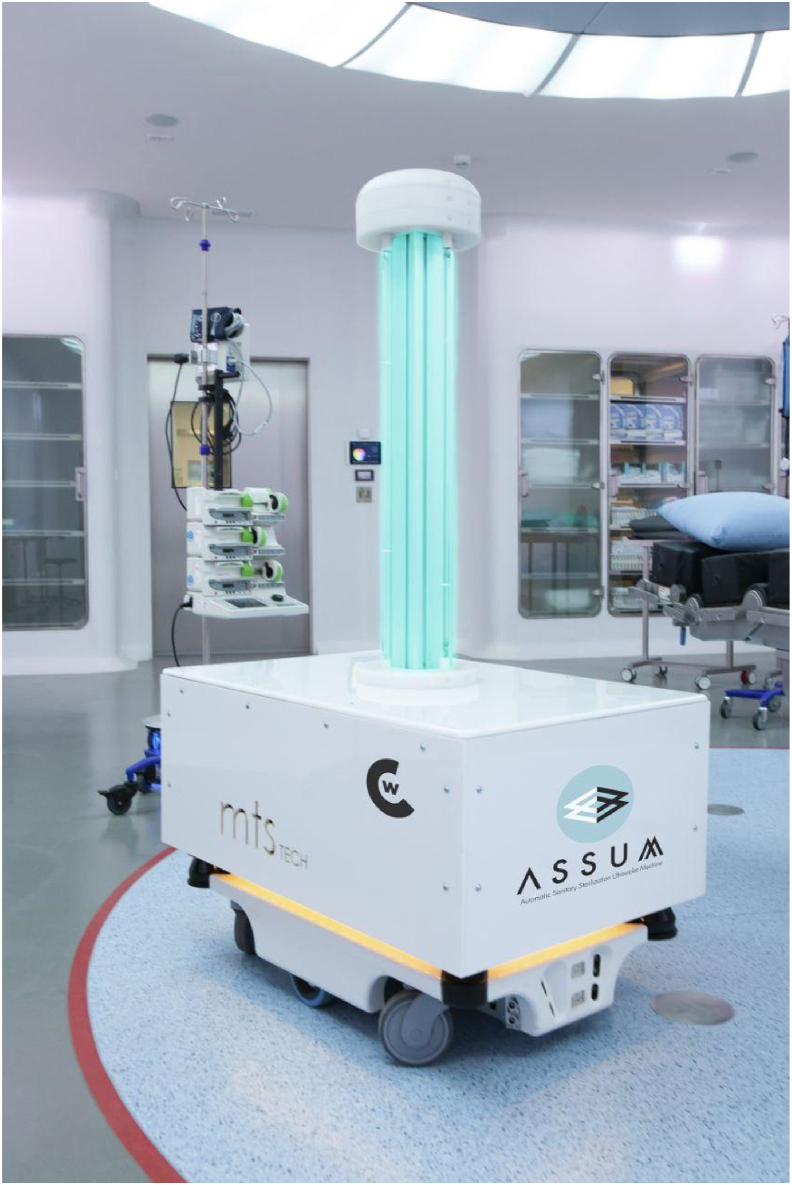
UV-C light robot: ASSUM (Autonomous Sanitary Sterilization Ultraviolet Machine).

The ASSUM (Autonomous Sanitary Sterilization Ultraviolet Machine) consists of a germicidal ultraviolet light unit which produces UV-C with a peak of 254 nm by 4.25 W low-pressure lamps installed on a programmable mobile robot with autonomous operation capacity. The dose of UV-C received at 1 meter is 1273.8 J/m^2^. The device has an octahedral base on wheels 90 cm long and 60 cm wide on which the four UV-C emitting lamps are installed vertically, giving a total height of 150 cm (Figure 1).

The study was carried out in six operating rooms and one intensive care unit at the Hospital Clínic de Barcelona, a 760-bed, tertiary-care university centre. This facility has specialized critical care units, solid organ (liver, kidney, pancreas and heart) and hematopoietic stem cell transplant services, and it is a reference centre for cardiac, orthopaedic and colorectal surgeries. The study was approved by the Hospital Clínic of Barcelona Ethical Committee for Research (HCB-2022-1099).

### Intervention

After a two-month period of designing and programming the route maps and adjusting the safety protocols by the technicians of the device and those of the Hospital, ASSUM was used on a daily basis from Monday to Friday during ten consecutive months (from January 1, 2023 to October 30, 2023) in the afternoon shift (15.30 h PM to 21 h PM) in the following clinical settings: a) the six operating rooms where cardiac, colorectal and orthopaedic surgeries were routinely performed. These theatres were terminally disinfected with ASSUM after routine manual cleaning on the afternoon of every working day whenever they were unoccupied; and b) an intensive-intermediate care unit (ICU) of 14 individual beds attending preferentially surgical patients with complex intra-abdominal procedures, including liver transplantation, and patients with gastrointestinal bleeding disorders. The 6 intensive care and 8 intermediate care rooms of the study unit are located in the same ward and patients are looked after by the same personnel team. ASSUM was deployed in rooms previously occupied by patients infected or colonized by a multi drug resistant (MDR) microorganism qualifying them for contact precautions (methicillin-resistant *Staphylococcus aureus*, vancomycin-resistant *Enterococcus faecium*, linezolid-resistant *Staphylococcus epidermidis*, any extended spectrum beta-lactamase producing enteric Gram-negative bacilli different from *Escherichia coli*, any carbapenemase-producing *Enterobacterales*, multiple-drug resistant *Pseudomonas aeruginosa* or *Acinetobacter baumannii* and *Clostridioides difficile*). ASSUM was used in empty rooms after discharge of the patients and routine terminal manual cleaning. In surgical theaters and ICU rooms, terminal cleaning and disinfection was done by using 20 mL per 8 L of water of a quaternary ammonium mixture of N- (3-aminopropyl)-N-dodecylpropane-1,3-diamine (51 mg/g) plus dodecyl-dimethylammonium chloride (25 mg/g) (Surfanius, Laboratoires Anios, Lezennes, France).

During the designing and programming period, route maps of all ICU and operating rooms were taken. The robot was programmed to stay during 1 minute at ≤1 meter of 6–8 points located around the bed (rooms) or surgical tables (theatres) and the medical devices and apparel or other surfaces highly touched by the patients or health care workers. ASSUM could be operated manually if unexpected objects were in the planned route or if the size of ICU rooms made difficult its autonomous functioning. Total planned effective operating time (UV–C light emission time) of ASSUM was 15 minutes per room or theatre. ASSUM was managed by a single technician operator specifically trained for this purpose.

No other new infection control practices were implemented in the Centre during the 2022–2023 study period.

### Measurements

The effectiveness of ASSUM in the operating rooms was measured by comparing the surgical site infection rates recorded from January to October 2023 with those that occurred during 2022 in patients undergoing the six procedures performed in the UVL-cleaned theatres. The selected procedures were coronary by-pass surgery, cardiac valve surgery, primary hip and knee prosthesis placement and elective colon and rectal surgery. These operations were selected because they are routinely monitored by the hospital's infection control team using a standardized protocol. According to this protocol, for orthopaedic prosthetic and colorectal surgery, a minimum of 100 consecutive interventions should be reviewed if the total number of procedures carried out at the centre is higher than 100 and all of them if the number is less than 100; for cardiac surgery, all the procedures must be monitored.

The participating ICU was chosen because it was the one with the highest incidence of MDR organisms requiring contact precautions in our centre. Data on the incidence of MDR microorganisms were prospectively collected as part of the routine task of the hospital's infection control team according to protocols that did not change during the study period (2022 and 2023). Efficacy was measured by comparing the incidence in clinical samples of MDR microorganisms possibly acquired in the ICU (those detected beyond 48 h of admission) during the intervention period (January to October 2023) with that recorded during the same period of the previous year.

In addition, an assessment of exposure to UV-C light and a quantitative study of the microbial environmental burden were carried out in all ICU and operating rooms. For UV-C measurements, commercial dosimeter disc stickers (Chemdye^R^ CD87-1000 Disinfection control adhesive labels, Terragene, Santa Fe, Argentina) were used that changed from a basal yellow colour to orange or red upon exposure to 500 mJ/cm^2^ or 1000 mJ/cm^2^, respectively. In the ICU rooms, between 7 and 8 self-adhesive labels were deployed along the planned route of ASSUM in a predefined numbered disposition including frequently touch surfaces (mechanical ventilation machine, pumps tower, head and foot bed boards, bedside table, cupboards, keyboards and at least one wall). In the operating rooms, due to their larger size, between 14 and 20 dosimeters were arranged on items including the operating table, anesthesia machine, vital signs recording machine or monitors, pumps tower, extracorporeal circulation machine (cardiac surgery), sonography machine, medication cart, chairs and tables of anesthesiologist or nursing work stations, and three of the room walls.

For the environmental microbiology study, standard 24 cm^2^ Rodac contact plates containing non-selective Trypticase Soy Agar with LTHT neutralisers (Lecithin, Polysorbate 80, Histidine, Thiosulfate) (Tiseleb S.L. Barcelona, Spain) were used to take samples from five predefined high touch surfaces in both the ICU (mechanical ventilation machine, pumps tower, right bedrail if it have a flat surface allowing full contact with the Rodac plate or the bed head or footboards otherwise, bedside table and the medication cupboard) and operating rooms (operating table, anesthesia machine, vital sign recording machine or monitors, pumps tower, medication cart or tables of anesthesiologist or nursing work stations). Two sets of 5 samples were taken, one after manual cleaning but before ASSUM application and another after ASSUM performance. **Up to ten colony morphotypes were picked and identified to the species level. We did not look for *Clostridiodes difficile*.** No specific validation of this procedure was performed for this study.

### Statistical analysis

Incidence of surgical site infections was expressed as percentage of events over the total number of procedures, and means (±SD) were used as measures of central tendency for quantitative variables. Incidence of multiple-drug resistance microorganisms were expressed as new detections of any of the target microorganisms per 10,000 patient-days of ICU stay. Chi-square test or Fisher exact test were used to compare proportions, with Bonferroni correction when doing multiple comparisons. For quantitative variables, Wilcoxon paired signed rank test was use to compare pre- and post-intervention bacterial counts; and Kruskal-Wallis one-way ANOVA was used to compare distances from the UV-C emitter among the three ranges of radiation dosage measured by UVL dosimeters. For computation of the 95% confidence interval of the difference between two incidence rates and associated p-value, a free online calculator was used [26]. Other computations were done by the SPSS statistical package version 22.

## Results

During the study period, a total 439 effective interventions were performed, 103 (23.4%) in the ICU and 336 (76.5%) in the operating rooms. Mean (±SD) effective duration of disinfection with ASSUM was 15.11 (1.93) minutes. In the surgical rooms, 226 (51%) interventions were done with the device operating automatically, accordingly with the predesigned route maps. In the remaining cases, some kind of manual operation was required, mainly by the presence of unplanned blocking objects in the programmed paths. Conversely, for the same reasons and giving the relative small size of the rooms, ASSUM had to be operated manually in the great majority (89%) of ICU interventions. There were no safety issues during the fully implemented intervention period.

### Surgical site infections

The number, types and rates of surgical site infections are summarized in Table I. The rates of surgical site infections for all procedures in the pre- and post-intervention periods were 8.67% (80/922) and 7.5% (61/813), respectively, a non-significant difference (dif. -1.17%; 95%CI= -3.74%, 1.43%, p=0.37). There were no significant differences between periods in the total rate of infections or in the rate of superficial or deep infections for any of the procedures under vigilance, either considered individually or grouped by specialty (orthopaedic surgery: 1.93% in pre- vs. 0.48% in post-intervention, difference: -1.44, 95%CI= -4.4%, 1–07%, p=0.18; colorectal surgery: 13.63% vs. 10.47%, difference: -3.16%, 95%CI= -10.03, 3.54, p=0.35; cardiac surgery: 9.64% vs. 9.59%, difference: -0.05%, 95%CI= -3.84%, 3.77%, p=0.97) or by clean procedures (orthopaedic and cardiac: 7.5% in pre- vs. 6.6% in post-intervention, difference: -0.91%, 95%CI -3.6%, 1.8%), p=0.51).

**Table I:** Number, type and rates of surgical site infections observed during the study period for the operations undergoing surveillance

Surgical procedure	No. infections/Total procedures 2022^a^ (%)	No. infections/Total procedures 2023^b^ (%)	Rate difference 2023-2022 (95% CI)	p
Hip arthroplasty	2/103 (1.94%)	0/102	-1.94% (-6.8%, 1.95%)	0.15
- Superficial	0	0		
- Deep/Organ-space	2 (1.94%)	0		
Knee arthroplasty	2/104 (1.92%)	1/103 (0.97%)	-0.95% (-5.83%, 3.59%)	0.56
- Superficial	2 (1.92%)	0	-1.92% (-6.74%, 1.93%)	0.15
- Deep/Organ-space	0	1 (0.97%)	0.97% (-2-67%, 5.29%	0.31
Colon	8/100 (8%)	13/152 (8.55%)	0.55% (-7.26%, 7.31%)	0.87
- Superficial	1 (1%)	3 (1.97%)	0.97% (-3.66%, 4.73%)	0.54
- Deep/Organ-space	7 (7%)	10 (6.57%)	- 0.42% (-7.79, 5.8%)	0.89
Rectum	16/76 (21.05%)	7/39 (17.94%)	-3.11% (-16.87%, 13.48%)	0.69
- Superficial	1 (1.31%)	1 (2.56%)	1.24 (-4.33%, 6.83%)	0.62
- Deep/Organ-space	15 (19.73%)	6 (15.38%)	-4.35% (-17.48, 11.88%)	0.56
Coronary by-pass with graft	35/251 (13.94%)	31/231 (13.41%)	-0.41% (-6.67%, 5.72%)	0.86
- Superficial	26 (10.35%)	22 (9.53%)	-0.84% (-6.23%, 4.66%)	0.76
- Deep/Organ-space	9 (3.58%)	9 (3.89%)	0.31% (-3.27%, 4.05%)	0.85
Coronary by-pass without graft	1/33 (3.03%)	3/32 (9.37%)	6.34% (-7.39%, 21.39%)	0.29
- Superficial	0	1 (3.12%)	3.12% (-7.61, 15.74)	0.3
- Deep/Organ-space	1 (3.03%)	2 (6.25%)	3.21% (-9.87%, 17.33%)	0.53
Valve substitution	16/255 (6.27%)	6/154 (3.89%)	-2.37% (-6.6%, 2.57%)	0.3
- Superficial	10 (3.92%)	3 (1.94%)	-1.97% (-5.37%, 2.06%)	0.27
- Deep/Organ-space	6 (2.35%)	3 (1.94%)	-0.4% (-3.38%, 3.43%)	0.78
Total	80/922 (8.67%)	61/813 (7.5%)	-1.17% (-3.74%, 1.43%)	0.37
- Superficial	40 (4.33%)	30 (3.69%)	-0.64% (-2.5%, 1.15%)	0.49
- Deep/Organ-space	40 (4.33%)	31 (3.81%)	-0.54% (-2.4%, 1.39%)	0.58

a January to December 2022.

b January to October 2023.

### Incidence of multiple-drug resistant microorganisms

From January to October 2022, 648 patients were admitted to the study unit for a total of 3125 patient-days; the corresponding figures in 2023 were 698 and 3546, respectively. Comparative incidence of MDR microorganisms possibly acquired during the first 10 months of 2022 and 2023 in the ICU are shown in Table II. During the intervention period (first 10 months of 2023) there was a numerically higher rate of MDR microorganisms possibly acquired during ICU stay than in the control period (118.4 vs 76.8 per 10,000 patient-days, p=0.087). This was due to a significant increase in the rate of acquisition of MDR Gram-negative bacilli in surveillance samples (rectal swabs) during the intervention period (78.9 vs. 38.4 per 10,000 patient-days, p=0.032). However, the incidence of positive clinical samples remained unchanged (39.4 per 10,000 patient-days in the intervention period vs. 38.4 per 10,000 patient-days in the control period, p=0.94).

**Table II:** Comparative incidence of multiple-drug resistant microorganisms possibly acquired during the first 10 months of 2022 (pre-intervention period) and 2023 (intervention period) in the ICU

Microorganism	Sample	2022 incidence per 10000 patient-days (no. Events)	2023 incidence per 10000 patient-days (no. Events)	Incidence rate difference (95% CI)	p
Gram-negative bacilli	All	67.2 (21)	112.8 (40)	45.6 (-0.38, 91.5)	0.052
	Rectal swab	38.4 (12)	78.9 (28)	40.5 (3.3, 77.8)	0.032
	Any clinical	28.8 (9)	33.8 (12)	5 (-21.9, 32)	0.71
ESBL-*K. pneumoniae*	All	6.4 (2)	25.3 (9)	18.9 (-0.5, 38.5)	0.056
	Rectal swab	3.2 (1)	22.5 (8)	19.3 (1.7, 37)	0.031
	Any clinical	3.20 (1)	2.8 (1)	-0.37 (-8.7, 7.9)	0.92
AmpC-producing Enterobacterales	All	19.2 (6)	14.1 (5)	-5.1 (-24.6, 14.4)	0.6
	Rectal swab	9.6 (3)	14.1 (5)	4.5 (-12.1, 21.1)	0.59
	Any clinical	9.6 (3)	0	-9.6 (-19.7, 0.6)	0.065
Carbapenemase-producing Enterobacterales	All	0	19.7 (7)	19.7 (4.1, 35.3)	0.01
	Rectal swab	0	19.7 (7)	19.7 (4.1, 35.3)	0.01
	Any clinical	0	0	0	-
*P. aeruginosa*	All	35.2 (11)	42.3 (15)	7.1 (-22.9, 37.1)	0.64
	Rectal swab	19.2 (6)	19.7 (7)	0.54 (-20.6, 21.7)	0.96
	Any clinical	16 (5)	22.5 (8)	6.5 (-14.6, 27.7)	0.54
Other Gram-negative bacilli	All	6.4 (2)	11.2 (4)	4.9 (9.5, 19.3)	0.5
	Rectal swab	6.4 (2)	2.8 (1)	-3.6 (-13.7, 6.6)	0.49
	Any clinical	0	8.4 (3)	8.4 (-1.7, 18.6)	0.1
Methicillin-resistant *S. aureus*	All	0	2.8 (1)	2.8 (-3, 8.7)	0.34
	Nares/Pharynx	0	2.82 (1)	2.8 (-3, 8.7)	0.34
	Any clinical	0	0	0	-
Linezolid-resistant *S. epidermidis*	All	3.2 (1)	0	-3.2 (-9, 2.6)	0.28
	Blood culture	3.2 (1)	0	-3.2 (-9, 2.6)	0.28
*C. difficile*	Feces	6.4 (2)	2.8 (1)	-3.6 (-13.7, 6.6)	0.49
TOTAL MR	All	76.8 (24)	118.4 (42)	41.6 (-0.61–89.4)	0.087
	Surveillance swabs	38.4 (12)	81.7 (29)	43.3 (19.8, 67)	0.024
	Any clinical	38.4 (12)	39.4 (14)	1 (-28.9, 31.1)	0.94

ESBL: extended-spectrum β-lactamase.

### Irradiation delivery study

Out of 182 measurements of UVL, 2 (1%) were below the threshold needed to change the basal yellow colour of the dosimeter, 100 (54.9%) changed to orange (500 mJ/cm^2^) and 80 (43.9%) changed to red (1000 mJ/cm^2^). The dosimeters that did not change colour were both at 1.3 meters from the light source. The mean (±SD) exposure distance of the orange dosimeters was 0.97 (0.08) meters and that of the red ones was 0.81 (0.07) meters. These differences between groups were all significant by an ANOVA Kruskal-Wallis test at the 0.05 level. All the dosimeters exposed to 1 meter or less changed colour. Of the 73 dosimeters exposed at <0.9 meters, 7 (9.5%) turned orange and 66 (90.4%) red; of the 96 exposed at >0.9 meters and ≤1 meter, 82 (85.4%) turned orange and 14 (14.6%) red, and of those exposed beyond 1 meter (maximum 1.3 meters), 2 (15.3%) did not change colour, 11 (84.6%) change to orange and none turned red (Table III).

**Table III:** Relationship between distance of the self-adhesive labels dosimeters to the UV-C light source and exposure

UV-C light dose (mJ/cm^2^)			Distance in meters			Total
			<0.9	0.9–1	>1	
	<500	Count	0^a^	0^a^	2^b^	2
		% within distance	0,0%	0,0%	15,4%	1,1%
	500	Count	7^a^	82^b^	11^b^	100
		% within distance	9,6%	85,4%	84,6%	54,9%
	1000	Count	66^a^	14^b^	0^b^	80
		% within distance	90,4%	14,6%	0,0%	44,0%
Total		Count	73	96	13	182
		% within distance	100,0%	100,0%	100,0%	100,0%

Each superscript letter denotes a subgroup of distance categories whose column proportions are not significantly different at the 0.05 level after Bonferroni correction.

### Environmental microbial burden study

Three of the 6 operating rooms were free of bacteria after manual cleaning and all 6 were free after ASSUM operation; mean (SD) pre-ASSUM and post-ASSUM bacterial counts were 0.4 (1.2) CFU and 0 (p=0.083). Isolates from the operating rooms were all commensals and when present their counts were <10 CFU per plate. All 14 rooms in the ICU produced positive Rodac plates after manual cleaning and 6 were completely free of bacteria after ASSUM operation. All ICU isolates were commensals from the skin or oral flora, except one strain of *S. pneumoniae.* Mean pre-ASSUM and post-ASSUM bacterial counts per plate were 4.57 (7.4) colony forming units (CFU) and 0.27 (0.8) CFU, respectively, a highly significant difference (p<0.0001). In four ICU rooms, at least one sample had a pre-ASSUM count >15 CFU per plate but none produced ≥50 CFU; in all post-ASSUM samples grew ≤5 CFU per plate.

## Discussion

The present study has shown that the deployment of an UV-C light emitting robot (ASSUM) in two services of a tertiary care centre (surgical theatres and ICU rooms) is feasible, ensures adequate delivery of germicidal UV-C light to target areas and reduces significantly the bacterial burden remaining on surfaces after manual cleaning, particularly in the critical care setting. However, it has not been possible to demonstrate that these effects translate into beneficial clinical outcomes such as a reduction in the rates of surgical site infections or a lower acquisition of MDR microorganisms in ICU patients.

Several UVL-emitting machines are currently in use for different disinfection purposes. Those installed on robots have the theoretical advantage of autonomous mobility that may shorten the total duration of the disinfection cycles, minimize the extension of non-exposed (“shadowed”) zones and even operate with total autonomy after charting appropriate routes. However, the demonstration of feasibility and efficacy of UV-C robots in hospital settings is quite scarce [22,24,25]. The present study has proven that the deployment of a specific kind of robot, ASSUM, in a tertiary-care hospital is feasible, although several practical issues are of note. Firstly, the “busy” nature of the facilities made the completely autonomous operation during working hours rather unrealistic due to the need to ensure adherence to strict safety measures to avoid personnel exposure to UVL. This meant that the procedure had to be performed under the continuous supervision of a trained operator. Secondly, despite programming the routes, there were in practice frequent impediments on the designed path that only human intelligence can solve on the spot. Both of these issues were particularly common in the ICU. We believe that in hospitals, specific trained personnel should be in charge of managing UVL robots.

Our 15 minutes total cycle of UV-C light emission apparently guaranteed an adequate dosage on exposed inanimate surfaces. All the self-adhesive labels located at less or equal than 1 meter of the UV-C lamps received at least 500 mJ/cm^2^ (500,000 μWsec/cm^2^), a dosage that has been shown to be optimal for most microorganisms, including *C. difficile* [2,22]. The microbiology study also confirmed the disinfecting capacity of the robot beyond that achieved by manual cleaning. The lack of translation of these intended targets of ASSUM into measurable beneficial clinical outcomes may have several explanations. Firstly, efficacy of any non-touch disinfection technique used as a complement of the regular terminal cleaning is expected to depend on the quality of the manual procedure. Some studies have observed a relationship between the incidence of ICU-acquired infections and the room microbial burden, with most of them occurring in patients cared in rooms with >5 CFU/cm^2^ [2,27]. Most experts agree that a total bacterial count of <2.5–5 CFU/cm^2^ on hand touch sites and <1 CFU/cm^2^ of non-commensals are appropriate thresholds for benchmarking [28]. When using standard Rodac plates (≈24 cm^2^), these counts match the criteria of others, consisting of ≤15 CFU/plate for operating rooms and ≤50 CFU/plate for the ICU [8,9]. In our microbial burden study, after manual cleaning, no sample exceeded these marks, only once in the ICU grew a non-commensal and no MDR microorganisms were detected. Secondly, both surgical site infections and acquisition of resistant microorganisms may depend on other factors besides environmental bioburden. In one study documenting an association of UVL disinfection with a reduction in the rate of surgical site infections, only clean operations were significantly improved [6]. In regards to MDR microorganisms and *C. difficile*, we did not observe differences in the acquisition rates in clinical samples, the primary outcome. However, more patients acquired the target microorganisms in screening rectal swabs during the intervention period. We think that in the study unit, transmission via hands of personnel and possibly the presence of other environmental reservoirs (sink traps) were more important than the bioburden on room surfaces. Lastly, it is possible that the schedule of interventions was not frequent enough to have a clinical impact. No all operating rooms could be disinfected every day from Monday to Friday because it was not uncommon that some of them were in use during the working hours of the ASSUM operator. In the ICU, interventions also depended on patient discharge during the afternoon shift.

The study has some drawbacks. In terms of design, it is a before-after study, and for the incidence of surgical site infections, particularly in clean procedures, the study was probably under-powered given the usual low rate of the selected outcome. In regards to the microbial burden study, although all morphologically different colonies were identified to the species level, the use of non-selective agar could have reduced the sensitivity of the procedure. In addition, we did not control for other potential confounders of the relationship of the environmental microbial burden with outcomes. However we consider it unlikely that during the study period there were substantial changes in patient characteristics. The study was carried out during the COVID-19 pandemic, however the study period was past the algid pandemic season, at a time when our hospital was functioning for all practical purposes under regular parameters and the study ICU was not specifically dedicated to COVID-19 during the study period. Moreover, according to data of the infection control team of our centre (data not shown) there were no changes in surgical techniques or duration of operations, adequacy of surgical antibiotic prophylaxis or compliance with hand hygiene in the ICU. We did not look for C. difficile in the environmental samples, in some studies it has been stated that *C. difficile* spores are harder to eradicate [29]. Lastly, the study findings may not be generalisable to populations with different demographics, healthcare settings, or regions. Therefore, the sample characteristics and context in which the study was conducted should be considered when applying the findings to other populations.

In conclusion, deployment of a particular UV-C light-emitting autonomous robot (ASSUM) in surgical theatres and rooms of an intensive-intermediate care unit of 14 individual beds at a tertiary-care centre is feasible and fulfilled the intended expectations of delivering adequate germicidal UVL to inanimate items and decreasing the microbial burden beyond what was achieved by the usual manual procedure. However, no impact was observed either on the rate of surgical site infections or on the incidence of acquisition of MDR microorganisms in clinical samples.

## Conflict of interest statement

None declared.

## Funding sources

This work was funded by Balvi Pte Ltd, d.b.a. Balvi Filantropic Fund, 8 Temasek Boulevard #32-04 Suntec Tower Three Singapore 038988 Singapore and Biancoblas SL, Barcelona (Spain) throughout the Fundació Privada Món Clínic, Barcelona (Spain).

## Credit author statement

**Sabina Herrera:** Conceptualization, Writing - Original Draft, Review & Editing.

**Ignasi Roca:** Data Curation, Formal Analysis, Investigation, Validation, Review & Editing.

**Ana del Río:** Investigation, Methodology, Review & Editing.

**Javier Fernández:** Visualization, Review & Editing.

**Cristina Pitart:** Data Curation, Formal Analysis, Investigation, Validation, Review & Editing.

**Isabel Fortes:** Investigation, Review & Editing.

**Blanca Torralbo:** Investigation, Review & Editing.

**Gemina Santana:** Investigation, Review & Editing.

**Romina Parejo-González:** Investigation, Review & Editing.

**Andreu Veà-Baró:** Software, Formal Analysis, Visualization, Review & Editing.

**Josep Maria Campistol:** Supervision, Funding Acquisition, Review & Editing.

**Mireia Aguilar:** Methodology, Validation, Review & Editing.

**Sergi Degea:** Investigation, Data Curation, Review & Editing.

**Climent Casals-Pascual:** Supervision, Conceptualization, Review & Editing.

**Alex Soriano:** Conceptualization, Supervision, Review & Editing.

**José A. Martínez:** Conceptualization, Project Administration, Data Curation, Supervision, Writing, Review & Editing.

This statement details the specific contributions of each author in the creation and development of the research project.
